# Online Coupling of Field-Flow Fractionation with Raman Microspectroscopy Enables the Advanced Study of Nanoplastics Directly in Food

**DOI:** 10.1021/acs.analchem.5c05137

**Published:** 2025-12-31

**Authors:** Stefano Giordani, Maximilian J. Huber, Isabel S. Jüngling, Andrea Zattoni, Barbara Roda, Pierluigi Reschiglian, Valentina Marassi, Natalia P. Ivleva

**Affiliations:** † Department of Chemistry “Giacomo Ciamician”, University of Bologna, 40129 Bologna, Italy; ‡ byFlow srl, 40129 Bologna, Italy; § INBB − Biostructures and Biosystems National Institute, 00165 Rome, Italy; ∥ Chair of Analytical Chemistry and Water Chemistry, School of Natural Sciences, 9184 Technical University of Munich, Garching 85748, Germany

## Abstract

The detection and understanding of the behavior of nanoplastics (NPLs) in complex (in)­organic systems is a growing concern and one of the major challenges in analytical chemistry today. Current analytical methods are limited in terms of sample flexibility and automation, often require laborious pretreatment, and usually only provide limited information about the presence of NPLs without assessing the behavior of the plastics in the matrix. Coupling an asymmetrical flow field-flow fractionation multidetector (AF4-MD) platform with Raman microspectroscopy (RM) represents a significant advancement in the field, offering a novel approach that combines the advantages of a highly flexible, automatable, and informative analytical system (AF4-MD) with a detector able to chemically identify NPLs (RM). Up to now, this pioneering technique has only been used to study different nanoparticles in an aqueous environment. Here, for the first time, we report the application of an AF4-MD-RM platform to detect NPLs in a real unprocessed matrix. The developed approach allowed for the separation, selective detection, and multiparametric characterization of milk components and NPLs (polystyrene, PS beads, 100–500 nm) in a short analytical time without sample pretreatment, while providing PS detection threshold values compatible with those of the currently exploited quantification approaches. These beyond the state-of-the-art results were proved with orthogonal techniques and highlight the game-changing potential of AF4-MD-RM for a straightforward detection of NPLs in complex matrices and the characterization of NPL-matrix interactions.

## Graphical abstract

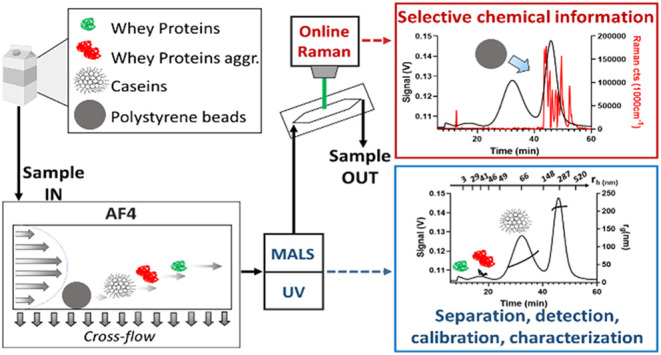

## Introduction

Nanoplastics (NPLs; diameter <1 μm) and microplastics (diameter between 1 and 5000 μm)^1^ (or MNPs, when referring to micro- and nanoplastics collectively) are plastic particles and fibers intentionally produced or formed via the fragmentation and degradation of previously released plastic debris in the environment.^2^ Their small size facilitates long-distance transport and cell permeability. Studies have demonstrated their ubiquitous presence in multiple matrixes such as water, air, food, and blood.^3–8^ They can impact ecosystems by leaching plastic additives and absorbing pollutants due to their high surface-to-volume ratio. Multiple studies have been published regarding the toxic effects of MNPs on animals, raising growing concerns about their possible effects on human health.^2^

Coverage of analytical approaches for MNPs has been extensively reviewed.^5,9–11^ Compared with the traditional visual observation approaches, chromatographic and spectroscopic techniques can improve the accuracy of MNP detection and provide information on their chemical composition. The disadvantage of the current most exploited chromatographic technologies (e.g., pyrolysis-gas chromatography-mass spectrometry, Py-GC-MS) is their limited applicability due to the high variations in sample preparation and the requirement of strictly pure samples with adequate concentrations.^5^ Moreover, they only provide information on the chemical composition and mass of MNPs, independent of particle-related information, i.e., number, size, and shape. Spectroscopic techniques (FT-IR and Raman spectroscopy) ensure accurate particle-based chemical identification and quantification of microplastics until roughly 10 and 1 μm, respectively.^9,12,13^ Furthermore, due to insensitivity to water, Raman spectroscopy can be online coupled to separation platforms enabling size-resolved physicochemical characterization of (plastic) particles down to 100 nm.^5,14,15^

Compared with microplastics, the study of nanoplastics presents significantly greater challenges, as reflected by the limited number of studies reporting their occurrence in complex matrices such as food. The separation of NPLs from these matrices is hindered by their compositional and density similarities to food components, complicating both chemical and physical isolation. Additionally, their nanoscale dimensions make NPLs more prone to chemical degradation during sample preparation, rendering digestion protocols effective for microplastics unsuitable.^16^ On the detection front, most established identification techniques for microplastics are only partially transferable to nanoplastics and often lack the sensitivity required to detect the low concentrations expected in real samples.^9^ Ideally, an optimal analytical method for NPLs should enable detection, chemical identification, size and shape characterization, and quantification across a broad size range, all while minimizing sample pretreatment.

A promising technique able to cover some of these features is asymmetrical flow field-flow fractionation (AF4), which is able to separate nanospecies in a broad range of sizes (1 nm–20 μm) according to their hydrodynamic radius (diffusivity).^17^ The separation takes place in a hollow channel, making the process gentle and allowing for a high degree of flexibility in terms of analytical conditions. This enables working under native conditions, minimizing sample pretreatment and thus obtaining more representative results.^18^ Additionally, since the technique is not destructive, it is possible to collect fractions of the separated samples for further analysis. Historically, the coupling with concentration detection (Absorption, dRI) and light scattering detectors has shown the ability of AF4 multidetection (AF4-MD) platforms to separate and characterize (size/shape, stability) NPLs standards^19,20^ as well as complex samples such as food^21,22^ and biological fluids.^23,24^

Currently, few pioneering studies have exploited AF4-MD to aid the analysis of NPLs in complex matrixes.^7,25–27^ However, in most of the studies, AF4-MD has been used as an intermediate step to simplify the samples, which are often subjected to digestion beforehand, before the selected fraction is directed to other offline techniques for chemical identification/quantification.

Recently, new possibilities in terms of AF4 detection were opened by the realization of online couplings of AF4 with nanoparticle tracking analysis (NTA)^28^ and Raman microspectroscopy (RM)^14,15^ allowing to evaluate NPL particles concentration and chemical composition, respectively. However, both techniques have only been tested for the analysis of NPLs under model conditions (water + surfactants), and their applicability to the analysis of NPLs in complex matrices has yet to be demonstrated.

This work provides the first proof of applicability of the AF4-RM online coupling to the analysis of NPLs in a real food matrix—milk. We developed an AF4-UV-MALS-RM method able to separate ultrahigh-temperature (UHT) skim milk components and NPLs across a broad range of sizes in saline conditions without pretreatment. AF4 combined with UV and MALS detection allowed the size characterization of the separated species, while RM provided the chemical identification and, hence, selective detection of polystyrene (PS) beads. Such a multiparametric detection enabled also to gather insights into the possible behavior of PS beads in the matrix. The results were confirmed by offline analysis of the collected fraction with orthogonal techniques (SEM-EDX and NTA), strengthening the potential of the presented approach.

## Materials and Methods

### Particles and Chemicals

Spherical PS particles with diameters of 500 nm (PS500) and 100 nm (PS100) were obtained from Applied Microspheres GmbH (Germany). Spherical 200 nm (PS200) and 300 nm (PS300) particles were obtained from BS-Partikel GmbH, Germany. Spherical 50 nm (PS50) particles were obtained from DukeStandards, Thermo Fisher Scientific (Massachusetts, USA). Sodium Chloride (NaCl, ≥99.0%) was purchased from Carl Roth GmbH (Germany), while Calcium Chloride (CaCl_2_, ≥99%) was purchased from Caesar & Loretz GmbH (Germany). Casein from bovine milk (No. C7078) was obtained from Sigma-Aldrich (Missouri, USA). All mobile phase solutions were prepared using Milli-Q water (Merck, Germany). Skimmed UHT Milk (Fat <5 g·L^–1^, Proteins = 36 g·L^–1^) was purchased from a supermarket after its distribution. Following preliminary experiments, no significant differences were observed throughout the study when using a 10-fold dilution of milk in the working mobile phase instead of undiluted milk. Therefore, this diluted solution was used in all analyses discussed in this study to allow greater flexibility in injectable volumes and to overcome limitations associated with the autosampler’s ability to accurately handle small sample volumes. Henceforth, this 10-fold diluted solution will be referred to simply as “milk” in this study.

### Asymmetrical Field-Flow Fractionation

AF4 was conducted using an AF2000 Multiflow FFF (Postnova Analytics GmbH, Germany) coupled with a UV Absorbance Detection System (Shimadzu SPD-20*A*/20AV) and a MALS instrument (PN3621 MALS Detector). Sample injections were handled by an autosampler (PN5300, Postnova Analytics GmbH, Germany). The AF4 channel exploited was 300 mm long, 350 μm thick, and with a 10 kDa cutoff regenerated cellulose membrane. NovaFFF version 2.2.0.1 software was used to control the instruments, set separation parameters, collect data, handle signals from the detectors (UV and MALS), and compute the radius and molar mass of particles during the measurements.

### Raman Microspectroscopy

A WITec *alpha300* confocal Raman microscope (Oxford Instruments, United Kingdom, Germany, equipped with a 532 nm DPSS laser, 40 mW at the sample) was used. The microscope was equipped with a water immersion objective from Carl Zeiss Microscopy GmbH, Germany (63×, “W Plan-Apochromat” series, N.A. = 1.0). The spectrometer (UHTS600 for VIS, 600 mm focal length) attached to the Raman microscope was equipped with a grating with 300 lines mm^–1^. A CCD camera (DU970N-BVF, Andor Technology Ltd., Northern Ireland) was used as a detector. The aluminum flow cell utilized to realize the AF4-RM coupling was described extensively in another work.^15^ All online measurements were performed using the time series mode with a 10 s spectrum integration over the whole time of particle injection and separation. The laser was switched off for 5 s every 55 s to preserve particle fractionation. The same setup was used for batch measurements, but the objective was exchanged for an EC Epiplan-Neofluar HD DIC (100×, N.A. = 0.9) from Carl Zeiss Microscopy GmbH, Germany. Dried caseins were analyzed on a glass surface, while an aqueous PS 500 nm was dried on a SEM aluminum holder. During both online and offline measurements, the Raman spectra were recorded in the range of 100 −3785 cm^–1^.

### Nanoparticle Tracking Analysis

To determine the hydrodynamic diameter and concentration of selected AF4 fractions, a ZetaView 230 (ParticleMetrix GmbH, Germany) was used. The samples were injected without further dilution into the system running in the light scattering mode. Each of the fractions taken in triplicate was analyzed in three repeated measurements using 11 positions in the sample cell. A system validation was performed before the measurements using a 100 nm PS bead size standard.

### SEM-EDX

A Sigma 300 VP Field Emission SEM from Carl Zeiss AG, Germany, in combination with a secondary electron detector and a Quantax XFlash 6|60 detector from Bruker Nano GmbH, Germany, was used to acquire SEM images and information on the elemental composition by EDX at an acceleration voltage of 10 kV and a working distance of around 9–10 mm. 2.5 μL of each sample was drop-casted on silicon wafers and air-dried at room temperature before analysis. EDX mapping and subsequent data analysis was performed using the ESPRIT 2.5 software from Bruker. The energy calibration of the EDX detector was performed by using a silicon wafer.

## Results and Discussion

### AF4-UV-MALS Method Development

Developing a method capable of separating multiple components of different nature (e.g., whey proteins, caseins, and NPLs), in a common representative environment, where their stability is unknown, requires rigorous optimization of the instrumental separation program, mobile phase composition, and sample pretreatments. To achieve optimal results, the order in which these parameters are optimized must follow a hierarchical decision-making workflow that prioritizes maximizing component stability in the channel (recovery) without relying on exogenous substances, such as surfactants, to enforce such stability. A schematization of the exploited approach is reported in the Supporting Information (SI, Figure S1).

Given the complexity of the system studied, a separation program (SI, Table S1) able to separate the components of the systems was first developed working in Milli-Q water, which was known from literature to grant good recovery for PS beads of various sizes.^29^ The setup allowed good separation of PS beads in a broad range of sizes (50–500 nm) and an acceptable separation of milk components (SI, Figure S2). The recovery for a milk and PS (300 and 500 nm) was >90% (SI, Tables S2 and S3).

To obtain more representative results of the real-life scenario of Milk-NPLs interaction conditions, it is necessary to employ a saline mobile phase (MP), as milk colloids are naturally dispersed in an aqueous saline medium, which plays an important role in milk colloids’ stability and behavior.^30–32^ Three mobile phases with increasing salt concentrations, ranging from 4.5 to 45 mM NaCl and 1 to 10 mM CaCl_2_, were evaluated (SI, Table S4). In the preliminary phase of this work, we examined the effect of different MP compositions on the structural integrity of milk samples and caseins using DLS (SI, Figure S3) and AF4 (SI, Figure S4), as well as on the AF4 separation performance (SI, Figure S5) and milk recovery in the AF4 channel (SI, Table S2). Milk samples diluted 1:10 in the saline media described in this study showed no significant differences in the average hydrodynamic radius determined by DLS (SI, Figure S3) or in the gyration radius of the AF4 peak corresponding to caseins (SI, Figure S4). Moreover, the presence of salt in the MPs reduced repulsive interactions between the membrane and milk components, improving the separation compared with pure water. Since no major differences in component size or separation efficiency were observed among the different saline conditions within the experimental time frame of the analysis, the mobile phase providing the highest milk recovery (MP-2, 22.5 mM NaCl + 5 mM CaCl_2_, recovery 82 ± 2%) was selected as the most suitable.

Fractionated milk (Figure 1, orange trace) exhibited three peaks having a hydrodynamic radius (*r*_h_) calculated from FFF theory^33^ centered around 3, 41, and 66 nm and a calculated gyration radius (*r*_g_) of 25 ± 1 nm and 58.0 ± 0.2 nm (for Peak 2 and Peak 3). According to such values and literature,^22,34^ the three peaks were identified as whey protein (Peak 1), whey protein aggregates and small/broken caseins (Peak 2), and caseins (Peak 3).

**Figure 1:**
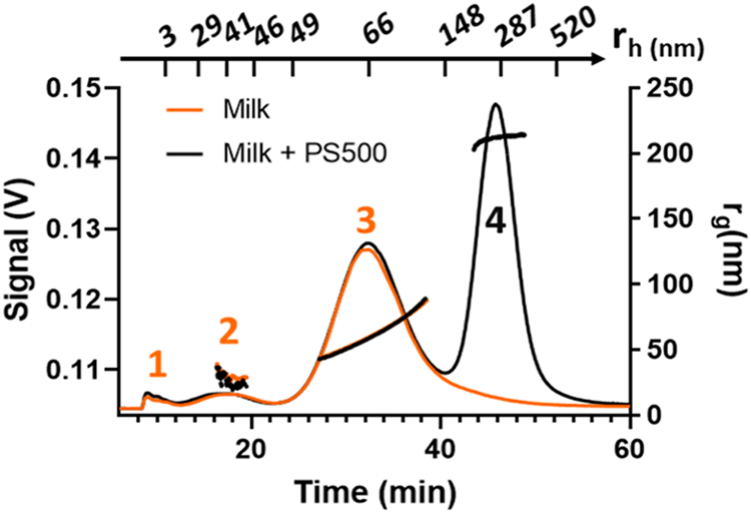
Representative UV (280 nm) profiles of milk (orange trace) and milk-PS500 mix (CPS500 = 275 mg·L^–1^, black trace) overlapped with the corresponding r_g_ values calculated from the LS signal. Additionally, r_h_ values calculated through separation theory are reported on the top of the figure.

Polystyrene beads were injected into all the saline mobile phases MP-1, MP-2, and MP-3 previously described (SI, Table S4), but no recovery was observed in any of them due to salt-mediated adsorption onto the membrane. Instead, the injection of a mixture made of milk spiked with PS500 beads to a final concentration of 275 mg·L^–1^, using the previously selected MP-2, highlighted the presence of a new peak compared to that of milk alone (Peak 4, Figure 1). The hydrodynamic radius (*r*_h_ ≈ 250 nm) and gyration radius (*r*_g_ = 210 ± 2 nm) of this peak were consistent with those of PS500. These results indicate that the beads remained stable within the channel in the presence of milk and that the analytical platform was capable of separating them even after a change in carrier medium.

The mix sample, stored at 4 °C, showed no significant variations over a 48 h period, suggesting that PS beads are stable in milk for at least 2 days. Interestingly, when a bovine serum albumin (BSA)-PS500 mixture (*C* ^PS500^ = 275 mg L^–1^) was injected, where the BSA concentration matched the whey protein content of the milk mixtures, PS recovery dropped to 0%. As reported by Kihara et al.,^35^ mixing negatively charged human serum albumin (HSA) with polystyrene beads in saline media promotes the formation of a soft protein corona that only minimally affects PS size and polydispersity. Under the tested conditions (MP-2), both caseins and BSA are negatively charged,^36,37^ and the PS500 particles likewise exhibited a negative ζ-potential (−26 ± 1 mV, measured by NTA). Therefore, the formation of a soft corona can be logically expected in the presence of either BSA or caseins. The different shielding abilities of the two coronas can be reasonably explained according to the size of the species involved. Caseins are more than 1 order of magnitude larger than BSA and likely provide more efficient steric stabilization of the PS particles compared to the smaller BSAs which fails to prevent membrane adsorption.^29^ However, a detailed elucidation of the underlying mechanisms would require additional experiments, which are beyond the scope of the present work. Overall, these experimental results highlight the stabilizing role of the caseins on PS beads within the AF4 channel. The recovery of both PS500 and PS300 when mixed with milk and injected using MP-2 as mobile phase was measured to be higher than 90% (SI, Table S3).

Based on these findings, 22.5 mM NaCl + 5 mM CaCl_2_ was selected as a successful working medium, along with the previously developed method.

### RM Offline Analysis

Regardless of the separation program exploited, according to their size, the NPLs considered in this study may fully or partially coelute with milk components (especially caseins). To chemically identify and selectively detect the two species, reference spectra of their respective standards were recorded, as described in Results and Discussion section (Figure 2). The spectrum of PS was characterized by significantly more intense signals and a better-resolved baseline compared with that of caseins, indicating a lower scattering efficiency for the latter. In particular, the common signal at 1000 cm^–1^, associated with aromatic ring breathing and representing the most intense peak for PS500, was over 40 times more intense than the corresponding signal (aromatic ring breathing from amino acid phenylalanine, Phe) in the casein spectrum. Furthermore, the comparison of the spectra revealed distinct characteristic signals for each species, whose monitoring over time could potentially allow selective elution monitoring of the species of interest.

**Figure 2:**
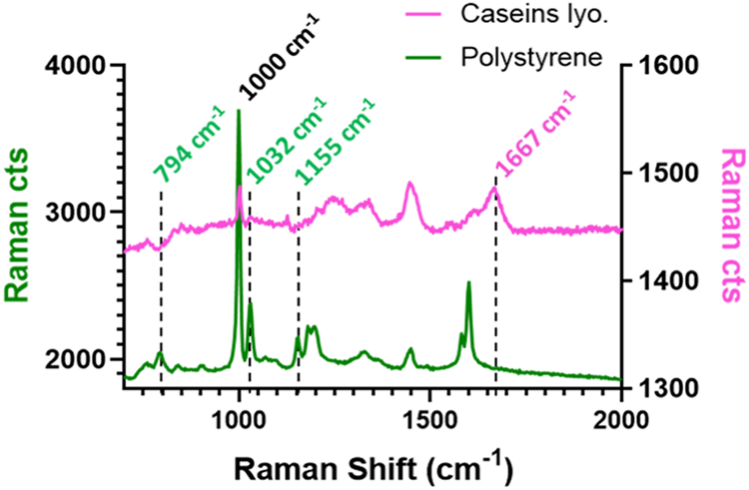
Polystyrene (green trace) and dried bovine milk casein (pink trace) reference spectra obtained by working in offline mode. The dotted lines highlight some of the characteristic wavenumbers which could be exploited for the selective monitoring of the two species.

### AF4-UV-MALS and RM Hyphenation

To assess the ability of the AF4-UV-MALS-RM platform to separate, identify, and characterize NPLs, the previously analyzed milk-PS500 mix was injected into the hyphenated setup. The results highlighted the ability of the Raman detector to trap PS beads and register the corresponding spectrum. By monitoring over time, the intensity of Raman shifts specific to PS (1032 cm^–1^), caseins (1667 cm^–1^), or common for the two (1000 cm^–1^, Figure 3A), it was also possible to observe the Raman signal only in correspondence with Peak 4. The average spectra for the latter matched perfectly with that of PS, confirming the previous peak attribution (Figure 3B). The small additional signal obtained at 14 min was also associated with PS and was probably originated from leftover particles in the flow cell or channel, which were flushed away during the analysis. Noteworthy, even by injecting a higher amount of milk (alone or mixed with the beads), it was not possible to observe particle trapping of the milk components. This is coherent with the structure of particulate milk components, which are softer, less dense, more heterogeneous, and more deformable than PS beads, and therefore, their trapping efficiency is expected to be much lower than that of solid NPLs spheres. A practical consequence of this phenomenon is the ability of the Raman setup, in the current working conditions, to act as a selective detection for PS and determine where PS particles distribute themselves in the fractogram even when coelution with milk components is observed.

**Figure 3:**
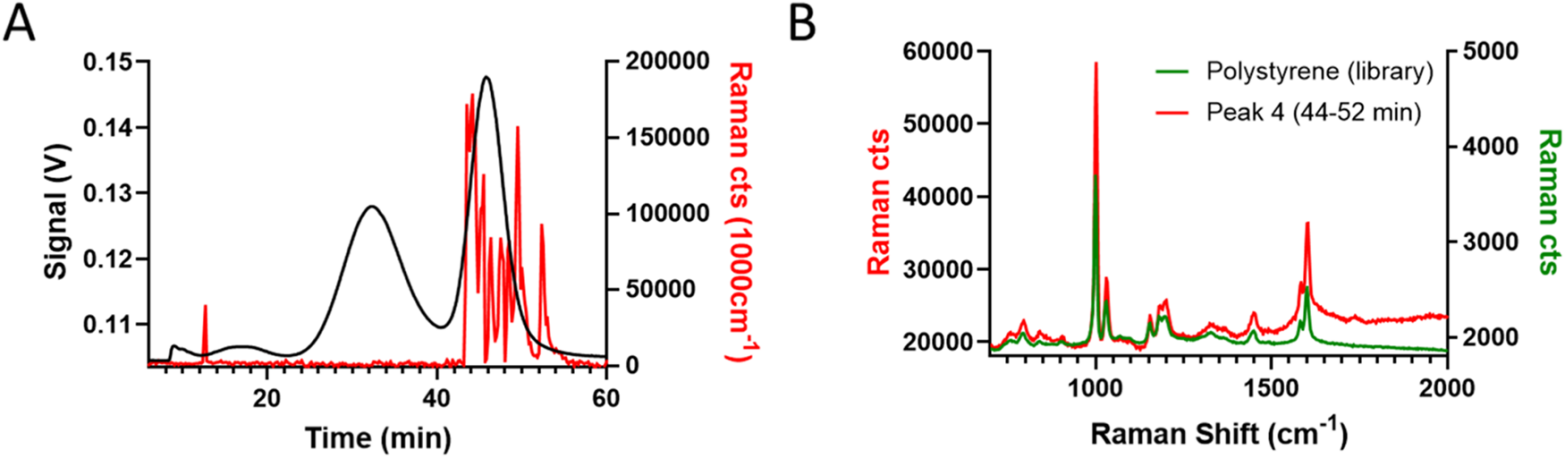
Representative outputs of online coupled Raman setup for the analysis of the milk-PS500 mix (*C* ^PS500^ = 275 mg·L^–1^). Comprehensive platform output. (A) Absorbance (280 nm, black trace) and Raman cts (1000 cm^–1^, red trace) fractograms of the mix. (B) Overlay between the reference spectra of PS and the average spectra of peak 4 (44–52 min). The comparison highlights a correspondence >95% between the two, confirming the chemical identification of Peak 4.

### Fractionation and Offline Characterization

The findings were verified with orthogonal techniques (NTA and SEM-EDX) through an offline analysis of Peak 4 for the 275 mg·L^–1^ mix obtained as an isolated fraction by collecting it after AF4 separation (Figure 4A).

**Figure 4:**
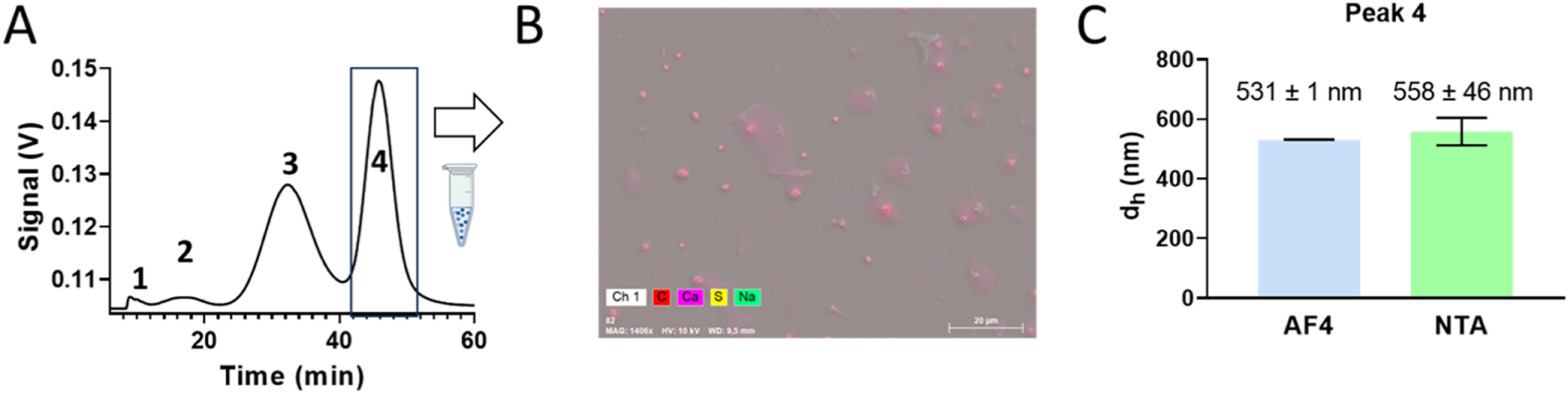
Offline analysis of particles from Peak 4. (A) Fractionation scheme highlighting the collection window of Peak 4 (42–52 min). (B) SEM + EDX image of particles from Peak 4. (C) Comparison between the hydrodynamic diameter calculated from NTA analysis and estimated from AF4 analysis.

SEM-EDX analysis revealed the presence of well-defined, nonaggregated, perfectly spherical carbon-based beads with a diameter of 500 nm (Figure 4B). NTA confirmed a fairly monodisperse population with a hydrodynamic diameter of 558 ± 46 nm, in good agreement with the MALS-derived size (531 ± 1 nm), considering the expected geometric relationship between *r*_g_ and *r*_h_ for solid spheres. Furthermore, the particle concentration estimated by NTA (approximately 10^7^ particles.mL^–1^) was consistent with the theoretical value expected taking into account PS500 recovery during AF4 separation.

Collectively, these orthogonal techniques validated the results obtained using our AF4-RM platform, further highlighting both the remarkable stability of polystyrene particles in the milk matrix and the minimal interaction between the sample and the separative system.

### Raman Threshold for PS500 and PS300 and Feasibility of Smaller-Size Entrapment via PS100 Analysis

Although the current Raman setup, due to the stochastic nature of particle trapping, is not yet capable of providing quantitative information on PS concentration, it was still possible to identify an indicative Raman Detection Threshold (RDT) for the analyzed PS beads. This parameter was defined as the minimum conditions (in terms of injected PS amount and PS concentration in the mixture) that produced a detectable Raman signal, indicative of PS contamination, in at least 50% of the injections, without reaching overloading conditions and thus preserving population separation. The RDT was estimated by monitoring the Raman peak at 1000 cm^–1^, the most intense shift associated with PS, and by performing six injections for each mix analyzed.

By varying both injection volume and sample concentration, the identified threshold condition for PS500 in the exploited instrument was 0.20 μg (20 mg·L^–1^) of injected PS500 (Figure 5A). This analytical approach was also successfully extended to 300 nm PS beads (PS300), where the system was able to both separate the particles from milk components and selectively detect their presence and evaluate their stability. The calculated gyration radius and the *r*_h_ values for PS300 suggested general stabilization of the beads within the milk matrix and the RDT for PS300 were found to be roughly around 0.24 μg (24 mg·L^–1^) (Figure 5B). As further evidence of the urgent need to develop robust MNPs detection strategies for milk safety, only a few studies have reported the presence of microplastics in commercial milk and dairy products.^38–40^ Investigations on NPLs have so far been limited to model samples prepared by dilution and/or spiking of milk (Table 1); therefore, no realistic concentration levels of NPLs in milk are currently available.

**Figure 5:**
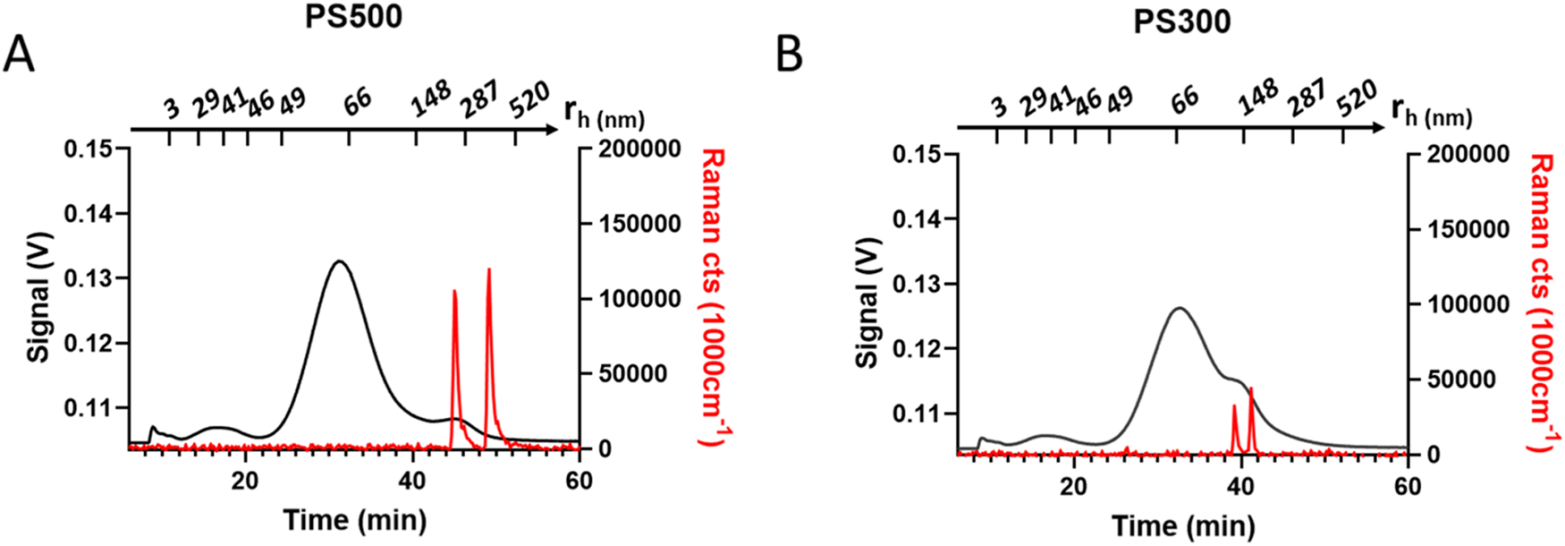
Platform output for the analysis of PS500 and PS300 mixes with milk around RDT conditions. (A) *m*_inj_^PS500^ = 0.20 μg, *C* ^PS500^ = 20 mg·L^–1^, (B) *m*_inj_^PS300^ = 0.24 μg, *C* ^PS300^ = 24 mg·L^–1^. The continuous black traces are associated with the absorbance signal (280 nm) while the continuous red traces are Raman cts (1000 cm^–1^). In both fractograms, the Raman signal is only visible at the small shoulder peaks, which elute at the retention time expected for particles of the assigned size, in agreement with retention theory and confirming this attribution.

**Table 1:** LOD/RDT of the Approaches Exploited to Detect NPLs in Milk Samples

technique	LOD/RDT	NPLs	size (nm)	refs
SERS sensor	100 mg·L^–1^	PET	50–300	^41^
SERS sensor	8.4 mg·L^–1^	PET	200	^42^
SERS sensor	50 mg·L^–1^	PS	400	^43^
SERS sensor	7.5 mg·L^–1^	PS	100	^42^
TGA-FTIR	61–128 μg	PS	1000	^44^
AF4-MD	0.20 μg (20 mg·L^–1^)	PS	500	this work
AF4-MD	0.24 μg (24 mg·L^–1^)	PS	300	this work

Overall, our results are comparable to those obtained by such approaches. However, unlike the latter, the presented approach provides a multiparametric characterization besides the mere NPL mass quantitation, does not require sample preparation, is potentially automated, and exploits mostly commercial instrumentations (furthermore, a commercial gold-coated version of the Raman flow cell is also available).

To detect 100 nm PS beads (PS100), which are more difficult to trap with the current flow cell, the Postnova Smart Stream Splitter module (PN1650, Postnova Analytics GmbH, Germany) was incorporated into the platform. This module preserved the separation achieved within the FFF channel while minimizing dilution and reducing the flow rate to the detectors (from 0.5 to 0.1 mL/min) improving particle trapping. Using this modified setup and injecting larger amounts of PS100, we were able to detect the presence of PS100 via the Raman detector. The limit condition at which the platform allowed the detection of PS100 contamination was 4 μg (123 mg·L^–1^) of injected PS particles (Figure S6). However, it was not possible to achieve visible separation of PS100 from caseins due to the higher sample amount injected and consequently higher coelution of the species. As a result, although the presence of PS100 in milk could be selectively confirmed, further characterization using MALS and standard separation theory was only partially feasible under these conditions, calling for further method development. Particle trapping, and therefore RDT, could be improved by using higher laser power or testing alternative laser wavelengths. For instance, a blue laser, due to its shorter wavelength, may be more effective for trapping smaller particles. Different surface coatings/materials of the Raman flow cell could be explored to further optimize the performance of 2D trapping. Finally, simply increasing the channel volume, although not directly enhancing resolution, may allow for higher injection amounts before overloading occurs, which could increase the concentration of NPL particles in the flow cell potentially improving the sensitivity of Raman analysis.

## Conclusions

For the first time, the ability of the AF4-RM online coupling to work directly on NPLs spiked to a real matrix under native conditions has been demonstrated. The developed platform combines the multiple advantages of AF4 in the analysis of real matrixes with a unique multidetector setup. This combination allowed a degree of sample pretreatment simplification, potential automation, and multiparametric characterization unmatched by the other analytical approaches exploited in NPLs analysis, while exhibiting comparable performances in terms of detection limit for PS500 and PS300. Such features provided unique representative insights regarding the stabilizing effect of caseins on PS beads in a saline environment; however, an analogous study employing a simulated milk ultrafiltrate solution^45^ as the mobile phase would be needed to confirm the phenomena. Although this work focused exclusively on monodisperse standard PS beads, the setup was able to detect PS even when significant coelution with milk components occurred (PS300). This demonstrates the feasibility of polymer-specific detection in a highly scattering and absorbing matrix. However, these experiments may not fully represent the analytical complexity expected for real milk samples, where nanoparticle populations may be broadly polydisperse and made of multiple different polymers, representing an additional analytical challenge. Previous studies have demonstrated the capability of AF4–Raman to discriminate nanoparticles made of different polymer types (PS, PMMA, PE) in water, including polydisperse samples down to 100 nm.^14,15^ Nonetheless, the direct transfer of these capabilities to milk and other component-heavy matrices poses different challenges. Accurate size characterization of the plastics may not always be applicable since MALS results are affected by coelution of multiple species, while retention theory assumes that particles are spherical and that there are no significant interactions between the sample and membrane. Furthermore, the behavior of different polymer types and particle sizes in the matrixes, and their impact on Raman detectability and trapping efficiency should also be taken into account by developing case-specific calibration tailored to the food matrix. Overall, considering the presented features of the setup and the high flexibility of AF4 as a technique, the translation of this approach to other matrices and NPLs is envisioned. The addition of NaN_3_ to the carrier to avoid the formation of biofilms in the channel^22,46^ while working with samples deriving from unprocessed matrixes possessing a living microbiota could also be considered. Furthermore, the implementation of chemometric tools to analyze and deconvolute the Raman spectra could help to discriminate analytes of interest in the case of simultaneous trapping of different species such as plastics and matrix components.^47^ Currently, the main limitations of the setup are connected to particle trapping, which becomes less probable as the concentration decreases, hampering the analysis of the NPLs. Although no clear limits are reported in the literature for NPLs in milk, the Raman Detection Threshold provided by the setup is currently likely too high. Reducing these limits to match the concentration range of NPLs typically found in water (roughly 0.04–52.3 μg·L^–1^)^48–50^ represents the main path to improving the setup. The most straightforward approach to achieve this is probably enhancing particle trapping, for example, by employing more powerful lasers (a laser power of 40 mW was used in this study), and exploring different laser wavelengths and different surface coatings/materials for the Raman flow cell. This could allow us to go beyond the current RTD. Future advancements should additionally focus on integrating complementary techniques, such as Nanoparticle Tracking Analysis,^15^ to achieve quantitative information on NPLs.

## Supplementary Material



## Notes

Data expanding on the methodological approach, mobile phase compositions, and their effect on sample elution and recovery, and on PS100 analysis (PDF)
