# Supplementary material for: Online Coupling of Field-Flow Fractionation with Raman Microspectroscopy Enables the Advanced Study of Nanoplastics Directly in Food
Source: Anal Chem. 2025 Dec 31;98(1):488–96. doi: 10.1021/acs.analchem.5c05137 (PMC12809650; doi:10.1021/acs.analchem.5c05137)
Supplement: Supplementary file 1 [file ac5c05137_si_001.pdf]

Supporting information (SI):

Online coupling of Field Flow Fractionation with Raman Microspectroscopy enables the advanced study of nanoplastics directly in food

Stefano Giordani [a,b,c], Maximilian J. Huber [d], Isabel S. Jüngling [d], Andrea Zattoni [a,b,c], Barbara Roda [a,b,c], Pierluigi Reschiglian [a,b,c], Valentina Marassi [a,b,c]*, Natalia P. Ivleva [d]*

[a] Department of Chemistry "Giacomo Ciamician", University of Bologna, 40129 Bologna, Italy
[b] byFlow srl, 40129 Bologna, Italy
[c] INBB – Biostructures and Biosystems National Institute, 00165 Rome, Italy
[d] Chair of Analytical Chemistry and Water Chemistry, School of Natural Sciences, Technical University of Munich, Garching 85748, Germany

Corresponding authors:
VM: valentina.marassi@unibo.it
NPI: natalia.ivleva@tum.de

Table of Content

Figure S1. Flow diagram schematizing the hierarchical and recursive approach exploited during method development. The recovery threshold was set to 70% in accordance with the international guidelines………………………………………………...………..………………………… **S3**

Table S1. AF4 separation method parameters……………………………………….………… **S4**

Figure S2. External calibration of the developed method in aqueous conditions. Representative UV fractograms (280nm) of injections of milk (red trace) and PS beads of various sizes (black traces)……….……………………………………………………………………………...**S4**

Recovery calculation………..……………………………………………………………... **S5**

Table S2. Recovery results for milk under all the tested conditions during mobile phase development.………………………….…………………………………………………... **S5**

Table S3. Recovery results for PS bead size (300nm and 500nm) alone and mixed with milk under all the tested conditions. The concentration of PS beads was 37 $mg.L^{-1}$, corresponding to that used in the mixes…….……………………………………………………………………………**S5**

Table S4. Mobile phases screened during method development. MP-2 was chosen as working mobile phase…………………………………………………………………………………**S5**

Figure S3. Average hydrodynamic radius determined by DLS of milk upon 1:10 dilution in the tested saline mobile phases. MP-1: 4.5mM NaCl + 1mM $CaCl_2$ pH = 6.7. MP-2: 22.5mM NaCl + 5mM $CaCl_2$ pH = 6.7. MP-3: 45mM NaCl + 10mM $CaCl_2$ pH = 6.7. Samples were stored at 4°C……....**S6**

Figure S4. Average gyration radius corresponding to the casein peak (Peak 3) obtained after AF4 separation of milk upon 1:10 dilution in the tested saline mobile phases. Each sample was analyzed while the AF4 system was conditioned with the same solution used for dilution. MP-1: 4.5mM NaCl + 1mM $CaCl_2$ pH = 6.7. MP-2: 22.5mM NaCl + 5mM $CaCl_2$ pH = 6.7. MP-3: 45mM NaCl + 10mM $CaCl_2$ pH = 6.7. ………………………………………………………………………………**S6**

Figure S5. Representative UV fractograms (280nm) of injections of the same amount of milk while working in different mobile phases. ……………………...……………………………………………………………... **S7**

Figure S6. The limit conditions at which the platform allowed the detected of PS100 contamination (m^{PS100}_{inj}= 4 µg, C^{PS100}= 123 $mg.L^{-1}$). The continuous black traces are associated to the absorbance signal (280 nm) while the continuous red traces are Raman cts (1000 cm^{-1}). ……………………...……………………………………………………………... **S7**

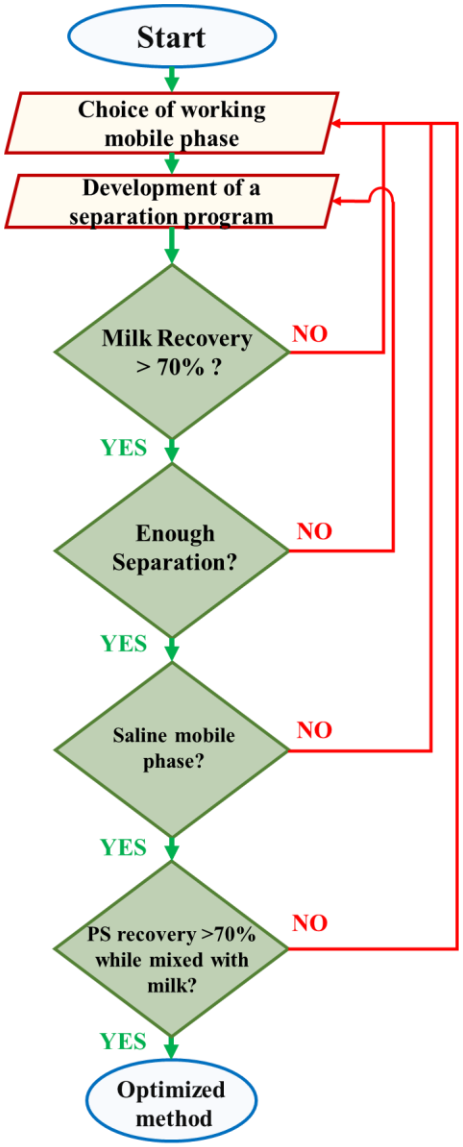

Figure S1. Flow diagram schematizing the hierarchical and recursive approach exploited during method development. The recovery threshold was set to 70% in accordance with the international guidelines.

Step	Flow	mL.min^{-1}	Time (min)
Whole method	Detector flow	0.5	80
Focus Step	Injection flow	0.2	7
	Cross flow	1.5	
Transition	/	/	1
Elution Step	Cross flow	1.5	2
		From 1.5 to 0.01 (power. 0.1)	50
		0.01	10
Rinse step	Cross flow	0	10

Table S1. AF4 separation method parameters.

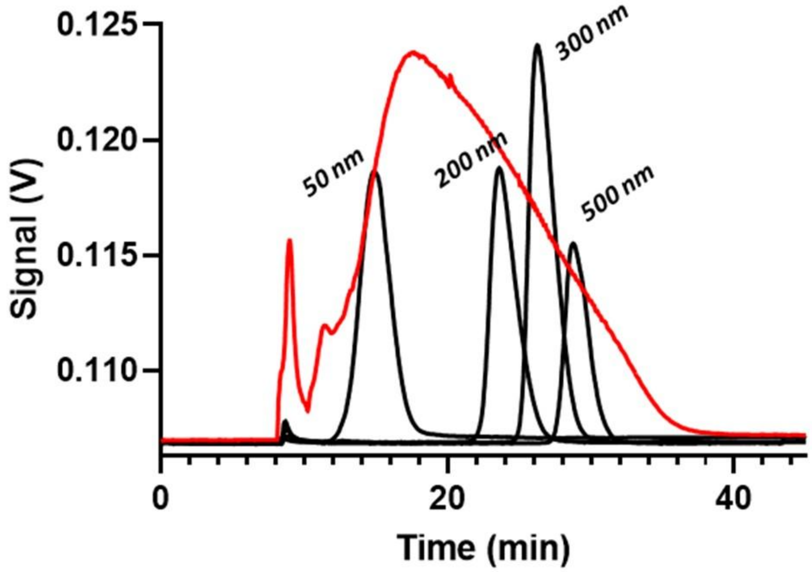

Figure S2. External calibration of the developed method in aqueous conditions. Representative UV fractograms (280nm) of injections of milk (red trace) and PS beads of various sizes (black traces).

Recovery calculation:

For all the samples (NPLs, milk and mixes) recovery was calculated as the ratio (%) between the total integrated area of the UV (280nm) fractograms associated to a 10µL sample injection with the optimized separation method (SM) and to a flow injection analysis (FIA).

$$R_{\%} = \frac{A_{SM}}{A_{FIA}} \times 100 \quad (1)$$

The recovery of PS beads while mixed in milk was calculated as the ratio (%) between the values obtained to the SM and FIA areas of the mixes (C^{PS}=37mg.L^{-1}) subtracting the areas of the SM and FIA of milk corrected according to the milk injection mass difference due to the forced settings of the injector.

$$R_{PS\ (in\ milk)} = \frac{A_{SM(mix)} - A_{SM(milk)}}{A_{FIA(mix)} - A_{FIA(milk)}} \times 100 \quad (2)$$

The recovery of casein alone could not be directly calculated, as the exact casein population present in the milk sample is not available as a pure, isolated standard. However, since caseins in skimmed bovine milk account for approximately 80% of the total protein content, the recovery of the colloidal fraction of skimmed milk can provide a reasonable estimation of the recovery of the isolated casein population. Zero recovery was defined when no clear peak was distinguishable from the baseline during SM.

	Recovery (%) for mobile phase development			
**Sample**	**PM-0**	**PM-1**	**PM-2 (chosen)**	**PM-3**
Milk	**91 ± 2%**	73 ± 2%	**82 ± 2%**	26 ± 5%

Table S2. Recovery results for milk under all the tested conditions during mobile phase development.

	Recovery (%) for PS beads in the tested conditions			
**Mobile phase**	**PS500**	**PS500 MILK**	**PS300**	**PS300 MILK**
PM -0	92 ± 1%	-	92 ± 5%	-
PM-2	0%	95 ± 1%	0%	93 ± 5%

Table S3. Recovery results for PS bead size (300nm and 500nm) alone and mixed with milk under all the tested conditions. The concentration of PS beads was 37 mg.L^{-1}, corresponding to that used in the mixes.

Mobile phase	Composition
MP-0	MilliQ water
MP-1	4.5mM NaCl + 1mM $CaCl_2$ pH = 6.7
MP-2	22.5mM NaCl + 5mM $CaCl_2$ pH = 6.7
MP-3	45mM NaCl + 10mM $CaCl_2$ pH = 6.7

Table S4. Mobile phases screened during method development. MP-2 was chosen as working mobile phase.

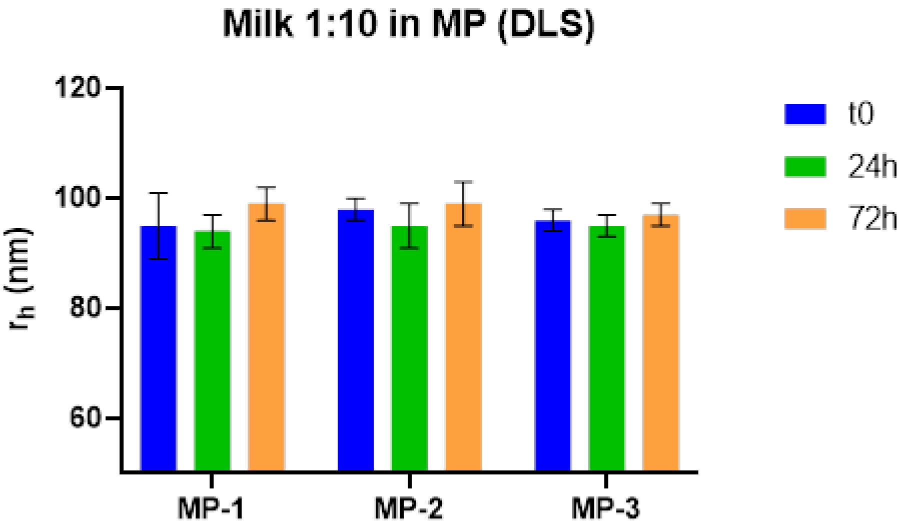

Figure S3. Average hydrodynamic radius determined by DLS of milk upon 1:10 dilution in the tested saline mobile phases. MP-1: 4.5mM NaCl + 1mM $CaCl_2$ pH = 6.7. MP-2: 22.5mM NaCl + 5mM $CaCl_2$ pH = 6.7. MP-3: 45mM NaCl + 10mM $CaCl_2$ pH = 6.7. Samples were stored at 4°C.

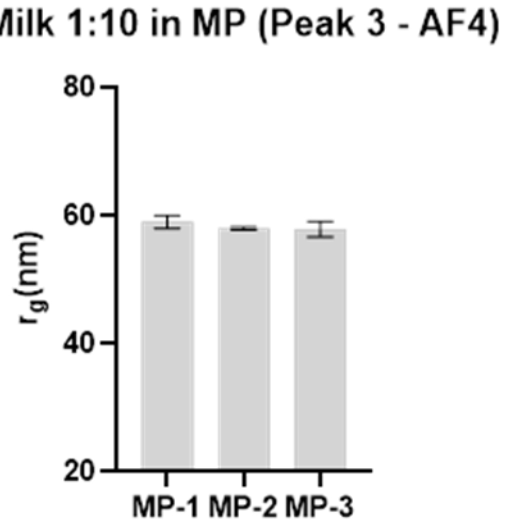

Figure S4. Average gyration radius corresponding to the casein peak (Peak 3) obtained after AF4 separation of milk upon 1:10 dilution in the tested saline mobile phases. Each sample was analyzed while the AF4 system was conditioned with the same solution used for dilution. MP-1: 4.5mM NaCl + 1mM $CaCl_2$ pH = 6.7. MP-2: 22.5mM NaCl + 5mM $CaCl_2$ pH = 6.7. MP-3: 45mM NaCl + 10mM $CaCl_2$ pH = 6.7.

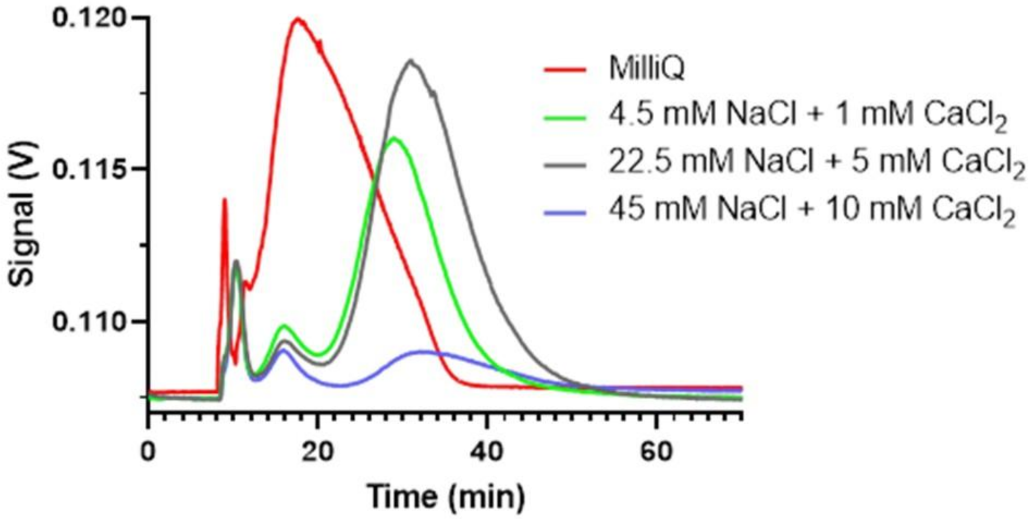

Figure S5. Representative UV fractograms (280nm) of injections of the same amount of milk while working in different mobile phases.

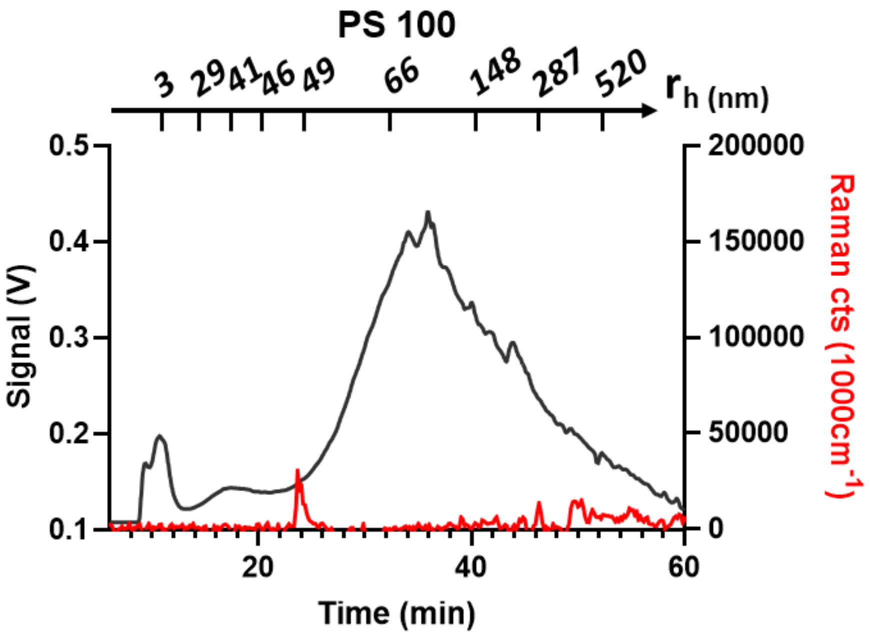

Figure S6. The limit conditions at which the platform allowed the detected of PS100 contamination (m^{PS100}_{inj}= 4 µg, C^{PS100}= 123 mg.L^{-1}). The continuous black traces are associated to the absorbance signal (280 nm) while the continuous red traces are Raman cts (1000 cm^{-1}).
